# Inflammation–HDL axis indices differentiate drug-naïve first-episode mania and recurrent mania from healthy controls: a covariate-adjusted study

**DOI:** 10.3389/fpsyt.2026.1780722

**Published:** 2026-03-05

**Authors:** Fatih Ekici, Rukiye Tekdemir, Ömer Bayırlı, Furkan Çınar, Mustafa Esad Tezcan

**Affiliations:** 1 Department of Psychiatry, Faculty of Medicine, Selcuk University, Konya, Türkiye; 2 Department of Child and Adolescent Psychiatry, Faculty of Medicine, Selçuk University, Konya, Türkiye

**Keywords:** atherogenic index of plasma, bipolar disorder, cardiometabolic risk, first-episode mania, HDL, inflammation, monocyte/HDL ratio, neutrophil/HDL ratio

## Abstract

**Background:**

Bipolar disorder (BD) is associated with immune dysregulation and cardiometabolic risk, yet low-cost biomarkers reflecting immune–lipid interactions across illness stages are not well defined.

**Methods:**

We compared 168 individuals with BD (69 drug-naive first-episode mania; 99 with a history of recurrent mania who were euthymic at assessment) and 60 controls (18–65 years). Diagnoses were established using SCID-5 and manic symptoms were assessed with the Young Mania Rating Scale (YMRS). Morning blood samples were used to compute the systemic inflammation response index (SIRI), neutrophil-to-high-density lipoprotein cholesterol ratio (NHR), monocyte-to-HDL ratio (MHR), low-density lipoprotein to HDL ratio (LHR), and the atherogenic index of plasma (AIP). Group differences were tested with general linear models adjusted for age, sex, and body mass index, applying false discovery rate correction across 12 indices.

**Results:**

YMRS was higher in first-episode mania than in euthymic recurrent patients (28.12 ± 3.10 vs 1.08 ± 1.18; p<0.001). After adjustment, SIRI, NHR, and MHR showed robust group effects; both BD groups had higher SIRI and NHR than controls, and MHR was elevated in both patient groups. LHR was higher in first-episode mania, whereas AIP was higher in the recurrent group compared with controls.

**Conclusion:**

Inflammation–HDL axis markers (SIRI, NHR, MHR) distinguish BD from controls independent of covariates, supporting immune–metabolic dysregulation and warranting prospective validation for risk stratification.

## 1 Introduction

Bipolar disorder (BD) is a severe, chronic psychiatric disorder affecting approximately 2% of the global population (1). It commonly emerges during adolescence or early adulthood and significantly impacts socio-professional functioning, contributing to a substantial reduction in life expectancy (2). Despite its profound impact and the urgent need for early intervention, a significant diagnostic delay of approximately nine years is common (3). This delay is attributable to factors including variable symptom presentation, a heterogeneous clinical course, multiple clinical subtypes, and the absence of reliable biomarkers (3). Current diagnostic reliance on subjective symptom assessment via standardized interviews hinders both early detection and a deeper understanding of BD’s pathophysiology (2–4). Therefore, the development of objective, accessible biomarkers is crucial.

Individuals with BD face a significantly increased risk of cardiovascular (CV) disease, contributing to a 10–15-year reduction in lifespan compared to the general population (5–7). Identifying the pathophysiological processes underlying this comorbidity is crucial for the early recognition and prompt intervention of high-risk individuals. While previous research has identified several factors contributing to increased mortality and morbidity in BD, systemic low-grade inflammation has emerged as a prominent potential etiological pathway shared with somatic illnesses (6, 8).

Growing evidence indicates that immune dysfunction plays a significant role in the pathophysiology of BD (9). Several studies have reported alterations in levels of proinflammatory cytokines, chemokines, C-reactive protein (CRP), brain-derived neurotrophic factor (BDNF), and oxidative stress markers in patients with BD (10–12). Although there have been a number of studies linking inflammation to BD (4, 9, 13, 14), blood biomarker studies are the most frequently used method of study; however, many of these biomarkers are hard to routinely collect or are expensive to measure, which may limit its largescale practical application in clinical practice (11). In contrast, the levels of neutrophils, monocytes, lymphocytes, and platelets are inexpensive and easily obtained via a complete blood count, providing an accessible means to assess systemic inflammation (11, 15). Numerous studies have investigated the Neutrophil-to-Lymphocyte Ratio (NLR), Monocyte-to-Lymphocyte Ratio (MLR), and Platelet-to-Lymphocyte Ratio (PLR) in relation to various psychiatric disorders, including BD (16). Additionally, novel composite indices like the Systemic Immune Inflammation Index (SII) and Systemic Inflammation Response Index (SIRI) integrate white blood cell subsets and platelets, reflecting the interplay between thrombocytosis, inflammation, and immunity (17). These indices have demonstrated predictive value for disease severity and prognosis in conditions such as ischemic stroke, CV disease, and pancreatitis (12, 17). Recently the utility of this index has been extended to psychiatric illnesses (11). However, while inflammatory markers have shown potential in predicting BD occurrence and prognosis, the association between immune dysfunction, BD, and elevated CV risk remains inconsistent across studies.

Furthermore, lipids and lipid metabolism are critical in the pathophysiology of neuropsychiatric disorders, including BD. This is attributed to the abundance of lipid species in the human brain, the close interplay between lipid metabolism and CV disease, and the lipid-altering effects of medications used to treat these disorders (18). There is also a strong connection between neuropsychiatric disorders, dyslipidemia, and the development of CV disease, whereby alterations in lipid metabolic pathways—particularly those involved in the immune system, such as arachidonic acid metabolism—are implicated (19). High-density lipoprotein (HDL), known for its antioxidant and anti-inflammatory properties, helps reduce pro-inflammatory cytokine release and facilitates cholesterol efflux, thereby alleviating inflammation (15). Consequently, ratios such as Neutrophil/HDL Ratio (NHR), Lymphocyte/HDL Ratio (LHR), Monocyte/HDL Ratio (MHR), and Platelet/HDL Ratio (PHR) have emerged as potential markers of systemic inflammation and oxidative stress across various disorders, including BD (19).

Recent advancements in CV risk prediction have characterized several atherogenic indices, which can provide insights into risk factors that are challenging to quantify with routine analyses and may reflect complex metabolic and clinical interactions among lipid fractions (20). Key atherogenic indices—including the Castelli Risk Ratio (CRR), Atherogenic Coefficient (AC), and Atherogenic Index of Plasma (AIP)—offer valuable insights into risk factors (20–22). CRR provides a simple summary of the balance between atherogenic and protective lipoprotein fractions; higher values indicate a more atherogenic lipid profile. Because it relies on routinely available total cholesterol and HDL-C, CRR can be readily derived in clinical settings. Overall, these findings suggest that lipids and biomolecules involved in lipid metabolism may serve as promising biomarkers for understanding BD pathophysiology and predicting CV risk.

However, dynamic changes in inflammatory and cardiometabolic risk–related indices across over time in BD remain debated. While peripheral inflammatory markers have been examined in BD, direct comparisons between drug-naïve First-Episode Mania (FEM) and Recurrent Manic Episodes (RME) using a comprehensive panel of routinely derived inflammatory and atherogenic indices are limited. Therefore, we compared drug-naïve FEM patients and euthymic RME patients with healthy controls (HCs) using routinely available blood parameters to calculate composite inflammatory indices and lipid/HDL-based and atherogenic risk indices, aiming to clarify whether these inexpensive markers differ between clinically defined groups in a cross-sectional design and to generate hypotheses regarding inflammation–HDL axis alterations and cardiometabolic vulnerability in BD.

## 2 Materials and methods

### 2.1 Subjects

The sample consisted of 168 BD patients (69 FEM patients, 99 RME patients) and (60) HCs, aged between 18 and 65 years. The cases came from the inpatients or outpatients who sought medical help in the psychiatric department of the Selcuk University, Department of Psychiatry, Mazhar Osman Mood Clinic. The HCs were volunteers recruited from the local community during the same period.

An experienced psychiatrist administered the Structured Clinical Interview for DSM-5- Clinician Version (SCID-5) to confirm the diagnoses of the patients. FEM group consisted of drug-naive patients who were experiencing their first manic episode and were subsequently hospitalized. RME group comprised patients with a diagnosis of BD for at least five years, who had a history of recurrent manic episodes and were currently being followed up as outpatients in a euthymic state. Participants were required to meet specific inclusion criteria; the FEM group consisted of individuals undergoing their first manic episode and individuals who have not used any psychiatric medication in the past year while the RME group included patients who had a BD diagnosis for a minimum of five years, a documented history of recurrent manic episodes, were receiving medication according to local standards for the treatment of BD and a current state of euthymia, indicated by Young Mania Rating Scale (YMRS) and Hamilton Depression Rating Scale (HAM-D) scores below (7) at the time of the study.

The exclusion criteria were as follows: (a) significant acute or severe illnesses, such as infections, autoimmune diseases, heart failure, head injuries, epilepsy, or tumors; (b) individuals undergoing anti-inflammatory treatment; (c) individuals with a history of psychoactive substance abuse within the past year; (d) patients with comorbid organic mental disorders; and (e) subjects with significantly elevated or decreased white blood cell (WBC) and platelet counts, which indicate current infection, to reduce the risk of including individuals affected by severe inflammatory diseases. Specifically, patients with leukocytosis (> (10) × 10^9 cells/L), leukopenia (< (4) × 10^9 cells/L), thrombocytosis (> 450 × 10^9 cells/L), or thrombocytopenia (< 100 × 10^9 cells/L) were excluded. The HCs underwent assessment using the SCID-5 to confirm the absence of psychiatric disorders. The exclusion criteria applied to the patient group were also enforced for the HCs.

This study was granted ethical approval by the Selçuk University Faculty of Medicine Local Ethics Committee (Decision Number: 2024/512).

### 2.2 Diagnosis and assessment of clinical characteristics of BD

After an initial diagnostic examination that included SCID-5, general demographic information and clinical parameters of the participants were noted. Physical comorbidities were confirmed by reviewing the patients’ previous medical history and medical records stored in our hospital’s electronic medical system. Diagnoses of mental comorbidities were made according to the DSM-V based on the subjects’ history of present illness and routine mental examination. Family history of mental disorders was assessed by asking the subjects or their accompanying relatives whether their first or second- degree relatives had mental disorders of any kind. All the above- mentioned interviews were conducted by trained psychiatrists in our study team. The symptomatic severity of BD was evaluated with the 17- item Hamilton Depression Scale (HAMD-17) (23) and the Young Mania Rating Scale (YMRS) (24).

### 2.3 Biochemical measurement

Data for WBC, neutrophils, lymphocytes, monocytes, platelets, cholesterol (CHO/TC), triglycerides (TG), HDL-C and low-density lipoprotein (LDL) were evaluated. Peripheral blood samples were drawn in the morning (between 7 and 9 a.m.). Blood sample assays were conducted by laboratory technicians who were blinded to the patients’ diagnoses. CRR was calculated as TC/HDL-C, AC was calculated as non-HDL/HDL (where non-HDL is the TC-HDL), and AIP was calculated as log10(TG/HDL-C), where the concentration of TG and HDL are in mmol/L. SII was calculated as the ratio of neutrophil counts to lymphocyte counts multiplied by the platelet count (platelet × neutrophil-to-lymphocyte ratio). The SIRI was calculated as the ratio of neutrophil counts to lymphocyte counts multiplied by the monocyte count (monocyte × neutrophil-to-lymphocyte ratio). The NHR was calculated by dividing the neutrophil value by the HDL value (neutrophil/HDL), the LHR was calculated by dividing the lymphocyte value by the HDL value (lymphocyte/HDL), the MHR was calculated by dividing the monocyte value by the HDL value (monocyte/HDL), and the PHR was calculated by dividing the platelet value by the HDL value (platelet/HDL). Additionally, the NLR was determined by dividing the neutrophil count by the lymphocyte count (neutrophil/lymphocyte), the MLR was derived by dividing the monocyte count by the lymphocyte count (monocyte/lymphocyte), and the PLR was calculated by dividing the platelet count by the lymphocyte count (platelet/lymphocyte).

We included a broad panel of routinely derivable indices to capture complementary domains relevant to BD and cardiometabolic vulnerability: (i) classical CBC-based inflammatory ratios (NLR, MLR, PLR) and composite indices (SII, SIRI), (ii) inflammation–HDL axis indices integrating immune-cell counts with HDL-C (NHR, LHR, MHR, PHR), and (iii) lipid-based atherogenic indices (AIP, AC, CRR). Because no single index is established as a gold-standard biomarker in BD, these analyses were designed as hypothesis-generating; multiplicity was addressed using Benjamini–Hochberg FDR correction.

### 2.4 Analysis

All analyses were performed using IBM SPSS Statistics (IBM Corp., Armonk, NY). Normality was evaluated using skewness and kurtosis. Continuous variables are reported as mean ± SD for approximately normal data and median (Q1–Q3) for skewed data; categorical variables are presented as n (%). All tests were two-sided, with p<0.05 considered statistically significant unless otherwise stated.

Clinical and illness-related variables were compared between the first-episode mania (FEM) and recurrent manic episode (RME) groups using independent-samples t tests or Mann–Whitney U tests for continuous variables, as appropriate. Categorical variables were analyzed using χ² tests or Fisher’s exact tests when expected cell counts were <5; corresponding test statistics (t, χ², or Z) and p values were reported.

Demographic variables and laboratory-derived indices were compared across RME, FEM, and healthy controls (HC) using one-way ANOVA or the Kruskal–Wallis test, as appropriate. Significant omnibus tests were followed by Tukey *post hoc* comparisons after ANOVA or Dunn’s test with Bonferroni correction after Kruskal–Wallis testing. Categorical variables were compared using χ² or Fisher’s exact tests, as appropriate.

Because groups differed in age and BMI, adjusted analyses were conducted using univariate general linear models (GLM) in SPSS. For each inflammatory or atherogenic index, the dependent variable was modeled with group (RME/FEM/HC) and sex as fixed factors and age (yas) and BMI as covariates. Type III sums of squares were used to estimate adjusted group effects. Partial eta squared (ηp²) was reported as an effect-size measure for the group term. Adjusted group-specific estimates were obtained as estimated marginal means (EMMs). Pairwise contrasts between groups (FEM–HC, RME–HC, and FEM–RME) were evaluated using Bonferroni-adjusted comparisons based on EMMs, and results are presented with 95% confidence intervals. For indices exhibiting right-skewed distributions, analyses were performed after natural log (ln) transformation to improve model fit and stabilize variance; for these outcomes, contrasts were expressed as geometric mean ratios with 95% confidence intervals. Indices that could take non-positive values (e.g., AIP and AC) were analyzed on the original scale.

To control for multiplicity across the set of indices, Benjamini–Hochberg false discovery rate (FDR) correction was applied to the family of adjusted group-effect tests; both raw p values and FDR-adjusted q values were reported.

## 3 Results

A total of 168 patients were included (FEM, n=69; RME, n=99). Compared with FEM, the RME group had an earlier age at onset, a longer illness duration, and a higher cumulative episode burden (total, manic/hypomanic, and depressive episodes). Psychotic symptoms, family history of psychiatric disorders, and comorbid medical conditions were comparable between groups. The similarly low prevalence of medical comorbidity across patient groups likely reflects the relatively young sample and the exclusion of participants with significant acute/severe medical or inflammatory illnesses; comorbidity was coded as the presence of at least one documented chronic medical diagnosis. First-episode polarity differed between groups, with a higher proportion of manic first episodes in RME. Although FEM participants were enrolled during their first manic episode, approximately half reported that their first lifetime mood episode was depressive, resulting in a near 50/50 distribution of first-episode polarity in FEM. These clinical characteristics are summarized in **Table 1**.

**Table 1:** Clinical characteristics of patient groups.

Variable	FEM (n=69)	RME (n=99)	Test statistic	p value
Age of onset (years)	25.62 ± 8.90	20.79 ± 5.32	-3.570	<0.001
Duration of BD (years)	1.35 ± 2.37	13.04 ± 6.67	-10.541	<0.001
Number of total episodes	1.73 ± 0.93	5.97 ± 4.52	-8.922	<0.001
Number of mania/hypomania episodes	1.00 ± 0.00	4.27 ± 3.60	-10.472	<0.001
Number of depression episodes	0.74 ± 0.93	1.96 ± 2.55	-4.478	<0.001
Psychotic symptoms during episodes, n (%)	36 (52.1)	53 (53.5)	0.371	0.547
First episode polarity, n (%)			13.28	0.001
Mania	35 (50.7)	67 (66.7)		
Depression	34 (49.3)	32 (32.3)		
YMRS score	28.12 ± 3.10	1.08 ± 1.18	68.94	<0.001
Family history of psychiatric disorders, n (%)^b^	25 (36.2)	41 (41.4)	0.702	0.402
Comorbid medical condition, n (%)	13 (18.8)	16 (16.1)	0.178	0.673
Pharmacotherapy (RME only), n (%)				
Aripiprazole	—	42 (42.4)		
Quetiapine	—	27 (27.2)		
Olanzapine	—	14 (14.1)		
Paliperidone	—	12 (12.1)		
Risperidone	—	3 (3.0)		
Lithium	—	58 (58.5)		
Valproic acid	—	47 (47.4)		
Lamotrigine	—	2 (2.0)		

Data are presented as mean ± SD for continuous variables and n (%) for categorical variables. Between-group comparisons used independent-samples t test (Welch correction when variances were unequal) for continuous variables and χ² test (or Fisher’s exact test when expected cell counts were <5) for categorical variables. Pharmacotherapy categories are not mutually exclusive; percentages may sum to >100%. ^b^Family history present. First episode polarity refers to the polarity of the first lifetime affective episode (depressive vs manic/hypomanic), not the index manic episode leading to enrollment.

BD, bipolar disorder; FEM, first-episode mania; RME, recurrent manic episode.

The study included 228 participants (RME, n=99; FEM, n=69; HC, n=60). Groups differed in age and BMI, whereas sex distribution was similar. In unadjusted analyses, several inflammatory and atherogenic indices differed across groups, generally showing higher values in patient groups compared with controls, with a stepwise pattern for AIP (HC < FEM < RME). Unadjusted group comparisons are presented in **Table 2**.

**Table 2:** Comparison of age, sex, BMI, and atherogenic/inflammatory indices across RME, FEM, and healthy controls.

Variable	RME (n=99)	FEM (n=69)	HC (n=60)	p value	Post-hoc
Age (years), mean ± SD	33.95 ± 7.73	26.93 ± 9.58	25.17 ± 7.83	<0.001	A > B = C
Sex, n (%)					
Female	47 (47.5)	36 (52.2)	33 (55.0)	0.633	
Male	52 (52.5)	33 (47.8)	27 (45.0)		
BMI, median (Q1–Q3)	23 (23–25)	22 (22–25)	21 (21–23)	<0.001	A > B > C
AC, median (Q1–Q3)	3.0962 (2.1923–4.3333)	2.5208 (1.6623–3.4625)	1.9781 (1.3624–2.7262)	<0.001	A > B = C
AIP, mean ± SD	0.5069 ± 0.3546	0.3016 ± 0.2850	0.1692 ± 0.2904	<0.001	A > B > C
CRR, median (Q1–Q3)	4.0962 (3.1923–5.3333)	3.5208 (2.6623–4.4625)	2.9781 (2.3624–3.7262)	<0.001	A > B = C
SII, median (Q1–Q3)	546.78 (380.06–838.33)	460.32 (175.22–652.58)	470.67 (371.76–594.42)	0.031	A > B = C
SIRI, median (Q1–Q3)	1.1723 (0.7749–1.6975)	1.1892 (0.6724–1.6862)	0.7628 (0.5393–1.0123)	<0.001	A = B > C
NHR, median (Q1–Q3)	0.1122 (0.0818–0.1581)	0.0991 (0.0754–0.1376)	0.0703 (0.0534–0.0907)	<0.001	A = B > C
LHR, median (Q1–Q3)	0.0541 (0.0416–0.0715)	0.0575 (0.0402–0.0747)	0.0399 (0.0311–0.0501)	<0.001	A = B > C
MHR, median (Q1–Q3)	0.0123 (0.0092–0.0186)	0.0142 (0.0099–0.0195)	0.0082 (0.0063–0.0108)	<0.001	A = B > C
PHR, median (Q1–Q3)	6.1053 (4.7917–7.6923)	5.7561 (4.2206–7.3931)	5.0730 (3.7455–6.2676)	0.010	A > C
NLR, median (Q1–Q3)	2.0415 (1.1875–2.8750)	1.6962 (1.4284–2.5748)	1.7336 (1.1326–2.0081)	0.028	A > C
MLR, mean ± SD	0.2486 ± 0.0896	0.2667 ± 0.1079	0.2181 ± 0.0801	0.013	B > A = C
PLR, mean ± SD	116.1513 ± 43.1097	109.9708 ± 49.7215	131.0540 ± 36.2804	0.021	C > A = B

Data are presented as mean ± SD for normally distributed variables and median (Q1–Q3) for non-normally distributed variables; categorical variables are n (%). Comparisons used one-way ANOVA (Tukey post hoc) for normally distributed variables, Kruskal–Wallis (Dunn–Bonferroni post hoc) for non-normally distributed variables, and χ² test for categorical variables. Groups: A=RME, B=FEM, C=HC. p<0.05 was considered statistically significant..

FEM, first-episode mania; RME, recurrent manic episode; HC, healthy controls; BMI, body mass index; SII, systemic immune-inflammation index; SIRI, systemic inflammation response index; NHR, neutrophil/HDL ratio; LHR, lymphocyte/HDL ratio; MHR, monocyte/HDL ratio; PHR, platelet/HDL ratio; NLR, neutrophil/lymphocyte ratio; MLR, monocyte/lymphocyte ratio; PLR, platelet/lymphocyte ratio; AC, atherogenic coefficient; AIP, atherogenic index of plasma; CRR, Castelli risk ratio.

To account for differences in age and BMI, we performed univariate general linear models adjusted for age, BMI, and sex and applied Benjamini–Hochberg FDR correction across the 12 indices. After adjustment and FDR correction, significant group effects (q<0.05) were observed for MHR, NHR, SIRI, LHR, and AIP. In Bonferroni-corrected pairwise comparisons, both patient groups showed higher SIRI and NHR than controls, and MHR was higher in FEM and RME than controls; LHR was higher in FEM than controls, while AIP was higher in RME than controls. Other indices did not retain FDR-significant adjusted group effects and should be interpreted as exploratory. Adjusted group effects and pairwise comparisons are shown in **Table 3**.

**Table 3:** Adjusted group effects and pairwise contrasts (GLM).

Marker	Scale	Group effect	FEM vs HC	RME vs HC	FEM vs RME
SII	ln	F(2,222)=2.481; p=0.086; q=0.119; ηp²=0.022	×0.98 (0.77–1.23); p=0.865	×1.24 (0.96–1.59); p=0.094	×0.79 (0.63–0.98); p=0.037
SIRI	ln	F(2,222)=7.342; p<0.001; q=0.003; ηp²=0.062	×1.42 (1.15–1.75); p=0.001	×1.49 (1.19–1.86); p<0.001	×0.95 (0.78–1.16); p=0.611
NHR	ln	F(2,222)=9.777; p<0.001; q<0.001; ηp²=0.081	×1.36 (1.16–1.60); p<0.001	×1.41 (1.19–1.66); p<0.001	×0.97 (0.84–1.13); p=0.686
LHR	ln	F(2,222)=5.649; p=0.004; q=0.010; ηp²=0.048	×1.28 (1.11–1.47); p<0.001	×1.16 (0.99–1.35); p=0.061	×1.10 (0.96–1.26); p=0.169
MHR	ln	F(2,222)=12.958; p<0.001; q<0.001; ηp²=0.105	×1.48 (1.27–1.72); p<0.001	×1.27 (1.08–1.49); p=0.004	×1.16 (1.01–1.34); p=0.040
PHR	ln	F(2,222)=0.157; p=0.855; q=0.855; ηp²=0.001	×1.02 (0.85–1.22); p=0.829	×1.05 (0.87–1.28); p=0.619	×0.97 (0.82–1.15); p=0.723
NLR	ln	F(2,222)=3.095; p=0.047; q=0.081; ηp²=0.027	×1.07 (0.92–1.24); p=0.373	×1.22 (1.04–1.43); p=0.015	×0.88 (0.76–1.01); p=0.078
MLR	ln	F(2,222)=2.222; p=0.111; q=0.133; ηp²=0.020	×1.16 (1.01–1.33); p=0.035	×1.10 (0.95–1.27); p=0.197	×1.05 (0.93–1.20); p=0.451
PLR	ln	F(2,222)=3.305; p=0.039; q=0.077; ηp²=0.029	×0.80 (0.67–0.95); p=0.012	×0.91 (0.75–1.10); p=0.333	×0.88 (0.74–1.04); p=0.140
AC	raw	F(2,222)=1.629; p=0.199; q=0.217; ηp²=0.014	Δ0.145 (-0.741–1.030); p=0.747	Δ0.776 (-0.163–1.716); p=0.105	Δ-0.632 (-1.465–0.202); p=0.137
AIP	raw	F(2,222)=5.800; p=0.004; q=0.010; ηp²=0.050	Δ0.068 (-0.035–0.172); p=0.197	Δ0.185 (0.075–0.295); p=0.001	Δ-0.116 (-0.214–−0.019); p=0.020
CRR	ln	F(2,222)=2.445; p=0.089; q=0.119; ηp²=0.022	×1.04 (0.92–1.18); p=0.535	×1.15 (1.01–1.32); p=0.041	×0.90 (0.80–1.02); p=0.089

Univariate general linear model (Type III) adjusted for age, BMI, and sex. Pairwise contrasts are reported with unadjusted p values and 95% confidence intervals and should be interpreted as supportive. For ln-scaled outcomes, effects are geometric mean ratios (×) with 95% CI; for raw outcomes, effects are adjusted mean differences (Δ) with 95% CI. q values are Benjamini–Hochberg FDR across the 12 group-effect tests. Markers with FDR q<0.05 are shown in bold to highlight the most robust adjusted group effects.

Abbreviations as in **Table 2**.

Adjusted group-specific estimated marginal means (geometric means for ln-transformed indices) are provided in **Table 4** and reflect the covariate-adjusted values underlying the contrasts in **Table 3**. Adjusted estimated marginal means are shown in **Table 4**.

**Table 4:** Adjusted estimated marginal means by group.

Marker	Scale	FEM	RME	HC
SII	Geometric mean (95% CI)	428.938 (365.938–502.784)	543.656 (470.939–627.602)	439.834 (366.663–527.606)
SIRI	Geometric mean (95% CI)	1.089 (0.945–1.255)	1.146 (1.008–1.303)	0.769 (0.654–0.905)
NHR	Geometric mean (95% CI)	0.103 (0.093–0.115)	0.107 (0.097–0.117)	0.076 (0.067–0.086)
LHR	Geometric mean (95% CI)	0.056 (0.051–0.062)	0.051 (0.047–0.056)	0.044 (0.040–0.049)
MHR	Geometric mean (95% CI)	0.014 (0.012–0.015)	0.012 (0.011–0.013)	0.009 (0.008–0.010)
PHR	Geometric mean (95% CI)	5.419 (4.797–6.121)	5.607 (5.022–6.259)	5.319 (4.627–6.116)
NLR	Geometric mean (95% CI)	1.832 (1.653–2.030)	2.084 (1.899–2.286)	1.714 (1.524–1.927)
MLR	Geometric mean (95% CI)	0.244 (0.222–0.267)	0.231 (0.212–0.251)	0.210 (0.189–0.234)
PLR	Geometric mean (95% CI)	95.985 (85.110–108.249)	109.610 (98.321–122.195)	120.253 (104.781–138.010)
AC	Adjusted mean (95% CI)	2.752 (2.153–3.351)	3.384 (2.842–3.925)	2.607 (1.921–3.293)
AIP	Adjusted mean (95% CI)	0.324 (0.254–0.395)	0.441 (0.378–0.504)	0.256 (0.176–0.336)
CRR	Geometric mean (95% CI)	3.502 (3.213–3.816)	3.878 (3.588–4.192)	3.361 (3.045–3.709)

Univariate general linear model (Type III) adjusted for age (yas), BMI, and sex. Values are estimated marginal means; geometric means are shown for ln-transformed outcomes. Group effects (including FDR q values) and pairwise contrasts are reported in **Table 3**..

Abbreviations as in **Table 2**.

## 4 Discussion

This study characterized peripheral inflammatory and cardiometabolic risk–related indices in BD by comparing drug-naïve first-episode mania (FEM) and recurrent manic episode (RME) patients with healthy controls (HCs). Inclusion of a drug-naïve FEM group is a key strength, as it provides insight into early-stage inflammatory and atherogenic profiles with reduced confounding by psychotropic medication. In unadjusted analyses, several atherogenic (AC, AIP, CRR) and inflammatory indices (including SII and SIRI, as well as HDL-based ratios) differed across groups, generally showing higher values in patient groups and a stepwise pattern for AIP (HC < FEM < RME). However, given between-group differences in age and BMI, we prioritized covariate-adjusted inference using univariate GLM models.

In adjusted models controlling for age, BMI, and sex, robust group effects were retained for indices integrating immune-cell profiles with lipid/HDL-related risk—most notably MHR, NHR, and SIRI, with additional adjusted differences for LHR and AIP. *Post hoc* comparisons indicated that both patient groups differed from controls for key inflammatory–HDL indices (SIRI and NHR), whereas stage-related contrasts were more selective: AIP was higher in RME than in HC, and LHR was higher in FEM than in HC. Collectively, these findings support the presence of an inflammatory–atherogenic signal in BD that is detectable in a drug-naïve FEM group and in euthymic recurrent patients. However, because this study is cross-sectional and groups differed substantially in age and BMI, these between-group differences should not be interpreted as within-person progression or “accumulation” over time; longitudinal studies are required to test temporal trajectory hypotheses, and residual confounding by unmeasured lifestyle and socioeconomic factors remains possible.

Our findings showed significantly elevated atherogenic indices (AC, AIP, CRR) in both FEM and RME groups compared to HCs. This aligns with extensive literature supporting the association between BD and increased CV risk (6, 7). Individuals with BD are known to have a higher incidence of CV disease and reduced life expectancy compared to the general population (7). Previous studies using various CV indices have also shown this elevated risk in BD patients (25, 26). AC, AIP, and CRR are novel risk calculators used to assess cardiovascular risk across various diseases. While a limited number of studies have used AC and AIP in BD, showing significantly higher levels in euthymic and acutely manic patients compared to controls, research using CRR in BD is lacking (15, 22). However, after covariate adjustment and FDR correction in our study, AIP retained a significant overall group effect, whereas AC and CRR did not. Accordingly, we interpret AIP as the most robust atherogenic signal in the adjusted models, while the unadjusted differences in AC/CRR may be partially attributable to age, BMI, and other confounders. Elevated atherogenic indices reflect lipid metabolism disturbances, specifically high triglycerides and low HDL levels—known risk factors for CV disease (19).

Increased risk indices in the FEM group indicate lipid metabolism dysfunction may be present even in a drug-naïve first-episode manic presentation, highlighting the need for research into its clinical implications (19, 27). One plausible mechanism is that acute mania may involve activation of innate immunity and an acute-phase response that can lower HDL-C levels and impair HDL’s anti-inflammatory functionality, while promoting hepatic production of triglyceride-rich lipoproteins. In parallel, stress-related neuroendocrine activation (HPA-axis and sympathetic activity) may alter lipid trafficking and lipoprotein remodeling even before chronic pharmacotherapy exposure. Finally, shared genetic liability and prodromal lifestyle disturbances (sleep loss, irregular diet, reduced activity, smoking) could contribute to early dyslipidemia and a more atherogenic lipid milieu in first-episode presentations. The higher unadjusted atherogenic indices observed in RME relative to FEM may reflect differences associated with greater illness burden and/or cumulative treatment and lifestyle exposures; however, progression of lipid profiles over time cannot be inferred from this cross-sectional design (27). Further research is needed to determine the relative contributions of illness characteristics, lifestyle factors, and long-term medication exposure (28, 29).

Another significant finding was the elevation of systemic inflammation markers in BD, particularly SIRI and inflammation–HDL ratios (NHR and MHR), supporting the role of immune dysregulation in BD pathophysiology (9). Research on SII and SIRI in BD remains limited. A recent large-cohort study reported higher SII, SIRI, and several HDL-based ratios in BD compared with healthy controls (12). In our **Table 2**, the unadjusted medians of SII and NLR appear slightly higher in healthy controls than in the FEM group; however, these differences are small and may reflect sampling variability and the ratio-based sensitivity of SII/NLR to modest within-normal-range shifts in neutrophils, lymphocytes, and platelets. Importantly, this descriptive HC>FEM median pattern did not persist in the covariate-adjusted GLM analyses (**Table 3**), where FEM did not differ from healthy controls for SII or NLR after FDR correction. By contrast, SIRI, NHR, and MHR showed the most robust adjusted group effects (q<0.05), suggesting that these indices may capture a clearer inflammation–HDL signal in this sample than SII/NLR. Classical ratios showed modest and inconsistent adjusted differences: NLR was higher in RME than controls, MLR showed a small FEM–HC contrast without a robust overall group effect after FDR, and PLR was lower in FEM than controls (11). Overall, these findings should be interpreted cautiously and warrant replication in larger, well-characterized cohorts.

The underlying mechanisms of CV disease susceptibility in BD are complex and not fully elucidated (26). Recent genetic and epidemiologic work suggests shared immunometabolic pathways between mood disorders and cardiovascular traits (30, 31). Immune dysfunction is a significant aspect of BD pathophysiology (9); atherosclerosis is an inflammatory disease, and novel immune modulatory treatments targeting atherosclerosis are under development (32). Therefore, the immune system likely plays a crucial role in both BD and CV diseases. Our cross-sectional findings are compatible with an association between an inflammation–HDL axis signal and atherogenic indices in BD (33, 34), but they cannot establish causality or mechanistic directionality. Future studies integrating longitudinal cardiometabolic outcomes, lifestyle and socioeconomic variables, and—when available—genetic information may help clarify whether these indices track clinically meaningful cardiometabolic risk trajectories in BD.

Routinely available complete blood count and lipid parameters enable inexpensive, scalable derivation of inflammatory and atherogenic indices, which is a practical advantage for real-world implementation. In our study, routinely derived indices—particularly those integrating immune-cell profiles with HDL-related risk and selected atherogenic measures—showed the most robust group differences after covariate adjustment. Given the modest effect sizes and the exploratory scope, these indices should be viewed as candidate adjunct markers for research-oriented phenotyping and cardiometabolic risk monitoring rather than tools for diagnostic classification or “biological stratification.” Before these indices can be considered for screening or clinical decision support, their diagnostic and prognostic utility (including discrimination, calibration, and incremental value beyond established risk factors) should be evaluated in prospective, longitudinal cohorts with standardized assessment of clinical state and treatment exposure.

Several limitations should be noted. First, the cross-sectional design precludes causal inference and limits conclusions regarding temporal relationships between BD course and inflammatory/atherogenic profiles. Accordingly, between-group differences should not be interpreted as evidence of progression or accumulation over time. Second, the single-center, relatively homogeneous sample may reduce generalizability. Third, although we adjusted for age, BMI, and sex, the groups differed markedly in age and BMI, and between-group differences in demographic and metabolic characteristics may still lead to residual confounding. Moreover, unmeasured factors (e.g., smoking status, physical activity, diet, sleep, and socioeconomic variables) could influence both inflammatory and lipid-based indices. Fourth, routinely derived indices can be affected by pre-analytical and state-related factors, including fasting status, time of blood sampling, recent infection or inflammatory conditions, and acute stress, which were not fully standardized in this design. Fifth, we did not perform diagnostic accuracy analyses (e.g., ROC/AUC) or assess incremental predictive value beyond established cardiometabolic risk factors. Finally, given the evaluation of multiple indices, the analyses should be considered partly exploratory despite the application of FDR control. Future studies should therefore include larger and more diverse cohorts, detailed characterization of medication exposure (type, dose, duration), lifestyle factors, and medical comorbidities, and employ longitudinal designs to examine prognostic value, state dependence across BD subtypes/episode phases, and potential utility for predicting treatment response. A key strength of the present study is the use of inexpensive, widely available routine blood parameters—including a drug-naïve FEM group—enabling a scalable approach to investigating inflammatory and cardiometabolic risk–related profiles in BD.

## 5 Conclusion

Routinely derived inflammatory and atherogenic indices differed across BD groups and healthy controls. After adjustment for age, BMI, and sex and FDR correction, the most robust differences were observed for SIRI, NHR, and MHR, with additional effects for LHR and AIP, suggesting an inflammation–atherogenic signal detectable in a drug-naïve first-episode mania group and in euthymic recurrent patients. These inexpensive markers warrant prospective, longitudinal validation—including diagnostic-accuracy and predictive analyses—to determine whether they provide clinically meaningful information for cardiometabolic risk profiling and research-oriented phenotyping in BD.

## Data Availability

The raw data supporting the conclusions of this article will be made available by the authors, without undue reservation.
